# Membrane perforation rate in lateral maxillary sinus floor augmentation using conventional rotating instruments and piezoelectric device—a meta-analysis

**DOI:** 10.1186/s40729-017-0114-2

**Published:** 2018-01-29

**Authors:** Corinne Jordi, Khaled Mukaddam, Jörg Thomas Lambrecht, Sebastian Kühl

**Affiliations:** Department of Oral Surgery, Oral Radiology and Oral Medicine, University Center for Dental Medicine, University of Basel, Basel, Switzerland

**Keywords:** Sinus lift, Conventional, Piezosurgery, Schneiderian membrane, Perforation, Ultrasound

## Abstract

**Objectives:**

Maxillary sinus augmentation (MSA) is a successful and predictable intervention with low complication rates. Perforations of the Schneiderian membrane may occur impairing the general success. The aim of this study was to compare the incidence of membrane perforations between conventional rotating instruments and piezoelectric devices in a meta-analysis.

**Material and methods:**

An electronic research on MEDLINE and PubMed database was performed evaluating the literature from 1980 till 2016. Meta-analysis was performed with the studies matching the inclusion criteria. The incidence of perforations between conventional and piezo during the lateral maxillary sinus floor elevation was determined, and forest plots and a *t* test for significance analysis were performed.

**Results:**

The search provided 377 articles of which 69 could be included. Selected non-randomised and non-controlled prospective and retrospective studies were incorporated. Conventional rotary instruments were associated with a perforation rate of 24%, the piezoelectric devices with 8% with statistically significant difference between both modalities (*p* < 0.05).

**Conclusion:**

Membrane perforations in MSA may be significantly reduced applying piezoelectrical devices for MSA.

## Review

### Background

Maxillary sinus augmentation (MSA) is a successful and predictable procedure to rehabilitate the atrophic edentulous posterior maxilla after postextractional pneumatisation of the sinus and bone loss with dental implants. Different approaches to elevate the maxillary sinus floor have been described and were originally introduced by Tatum [1, 2]. The lateral approach provides drilling a window in the lateral sinus wall for access to the Schneiderian membrane. This may be performed in terms of a total osteotomy or by drilling a trap door. Then, the Schneiderian membrane is carefully separated and elevated from the bony sinus floor and bone or bone grafting materials are applied to fill the new space between the membrane and bony sinus floor. This approach, however, is limited by the occurrence of Schneiderian membrane perforation while drilling the bony window in the sinus wall or separating the Schneiderian membrane from the bony floor. Schneiderian membrane perforation is the most frequent complication in MSA with an incidence of 7–44% [3, 4]. Two principal different techniques acceding and elevating the Schneiderian membrane are described. The conventional approach is performed applying rotary instruments in osteotomy which represents a risk for membrane perforation [5, 6], followed by the manual elevation of the membrane with hand instruments (special sinus lift kits). Alternatively, piezoelectric devices as proposed by Torella [7] and Vercelotti [8] may be applied for osteotomy and membrane preparation. Piezoelectric devices are specially designed for osseous surgery and use low-frequency ultrasonic vibrations. The amplitude of the micro vibrations allows a precise cut of bony structures without damaging the soft tissue [9]. Piezosurgery is being increasingly used in implant surgery, and the question rises whether the incidence of membrane perforations may be reduced using piezoelectric devices for MSA. Several cases are described, and many studies report on the occurrence of membrane perforation during MSA. However, only a few meta-analysis compare the incidence of membrane perforations associated to conventional (rotational) instruments and piezoelectric devices so far. There exist two reviews with a similar objective as ours (conventional versus piezosurgery device), one review which compared conventional sinus lift with four alternative techniques including piezosurgery and at least the review of Esposito analysing the study of Rickert et al. [10].

Atieh et al. [11] examined the intra- and postoperative events associated with the use of piezoelectric devices and conventional rotary instruments for lateral MSA in a systematic review. They included four studies with 178 lateral MSA in 120 participants. The meta-analysis did not show any significant difference between the two surgical techniques. Stacchi et al. [12] analysed the occurrence of intraoperative complications during sinus floor elevation with lateral approach and their correlations with the technique adopted by surgeons. They included 21 RCTs and 11 prospective CCTs. Rotary instruments, piezoelectric osteotomes, and manual bone scrapers were used to perform the lateral antrostomy. They found that ultrasonic devices and bone scrapers had a lower incidence (10.9 and 6.0%) of membrane perforation compared with that of rotating instruments (20.1%). They concluded that the thinning of the lateral wall of the sinus by using ultrasonic instruments or bone scrapers seemed to reduce the incidence of accidental sinus membrane perforations.

Geminiani et al. [13] assessed the difference in the incidence of intraoperative and postoperative complications between the conventional and alternative surgical techniques, during sinus floor augmentation surgery. This meta-analysis included 11 articles, while all compared the incidence of complications in conventional lateral window sinus augmentation surgery versus alternative techniques (osteotome: five articles, piezosurgery: four articles, sonic surgery: one article, trephine: one article). They found no statistically significant difference and concluded that the use of alternative techniques does not significantly reduce the incidence of intraoperative perforation of sinus membrane. Esposito et al. [14] researched in their review the beneficial or harmful effects of bone augmentation compared to no augmentation when undertaking a sinus lift procedure. They referred to the trial of Rickert, who undertook the comparison of rotary instruments versus piezosurgery to open a lateral window in the maxillary sinus, and found no evidence for the superiority of piezosurgery. This manuscript is a potential update exclusively on membrane perforation rate in lateral sinus augmentation procedures using conventional rotary or piezoelectric devices.

### Material and methods

The database PubMed and the US National Library of Medicine were screened from January 8, 2012, to January 6, 2016, for potential studies reporting on membrane perforations during MSA from 1980 till 2015. The search was conducted independently and in duplicate by two authors (MK and JC). The following search terms were used:

MeSH Terms:

1. Piezo-surgery
2. Ultrasound
3. Ultrasonic Osteotomy
4. Maxillary Schneiderian Membrane Perforation
5. Sinus Perforation
6. Maxillary Sinus Augmentation Complications
7. Lateral Sinus Osteotomy
  Cross-references:
8. Piezo-surgery AND Sinus Floor Elevation
9. Piezo-surgery AND Sinus Lift
10. Piezo-surgery AND Maxillary Sinus Grafting
11. Piezo AND Maxillary Sinus Lift
12. Piezoelectric Bone Surgery AND Sinus
13. Piezo-surgery AND Maxillary Sinus Augmentation Complications
14. Piezo-surgery AND Schneiderian Membrane Perforation
15. Ultrasound AND Sinus Augmentation
16. Ultrasound AND Sinus Lift
17. Ultrasonic AND Sinus Lift
18. Ultrasonic Osteotomy AND Sinus
19. Ultrasonic AND Schneiderian Membrane Perforation
20. Sinus Elevation AND Conventional

**Fig. 1:**
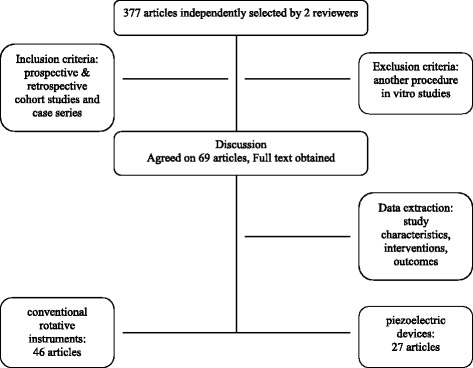
Result of the search strategy and included and excluded studies

Included were all studies reporting on the amount of membrane perforations during MSA by the lateral approach. Prospective and retrospective cohort studies and case series were also included. Both studies with split-mouth design and also studies without control group were also included. Excluded were studies describing any other procedure than the lateral approach for MSA, missing information on the occurrence of membrane perforation and in vitro studies. Titles and abstracts of the searches were initially screened for possible inclusion. After analysis of the abstracts, full-text evaluation was performed. Any disagreement was resolved by discussion between the authors (Fig. 1).

The following data were extracted:

1. Study characteristics: title, authors’ name, year of publication, study design, number of sinus floor elevations (SFE)
2. Interventions: the use of piezoelectric devices or rotary instruments for SFE
3. Outcomes: number and percentage of membrane perforation

#### Data synthesis

For each study, the sample size was determined and the event rate (e.g. perforation or not) was noted in an Excel sheet. Then, a meta-analysis was performed using the Comprehensive Meta-Analysis (Version 3) (Biostat, Englewood, USA) applying the sample size of each study and the event rate (e.g. perforation of the membrane or not). The software calculates the suggested effect of the operation technique (piezo or conventional) on the specific event (perforation of the membrane). This way, the raw data were weighted on the sample size for significance analysis. Applying the weighted data forest plots were calculated, indicating the weight and the 95% confidence interval. Additionally, the random effect was calculated representing the average of all studies in the respective groups (piezo and conventional).

**Fig. 2:**
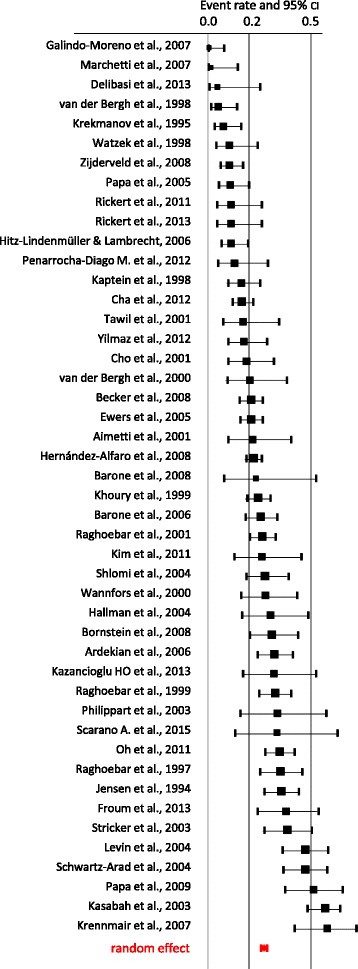
Forest plot of random effects meta-analysis of the incidence of Schneiderian membrane perforation using conventional rotative instruments. The weighted average for the incidence rate of Schneiderian membrane perforation was 24%

**Fig. 3:**
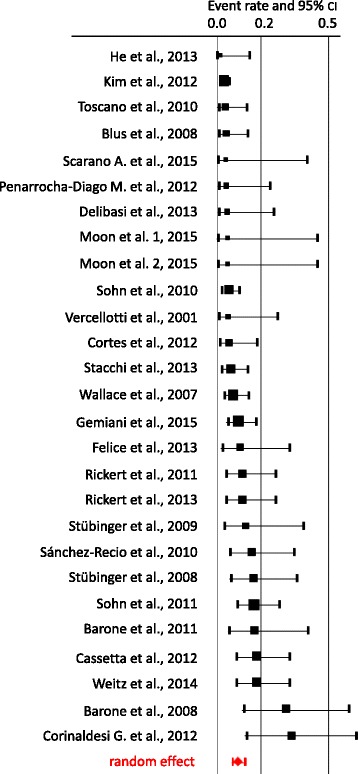
Forest plot of random effects meta-analysis of the incidence of Schneiderian membrane perforation using piezoelectric devices. The weighted average for the incidence rate of Schneiderian membrane perforation was 8%

Finally, a significance analysis was performed between both groups in terms of a *t* test. The significance level was set at *p* < 0.05 (Figs. 2 and 3).

### Results

#### Description of studies

Abstracts of 377 articles were screened. Of these, 69 studies matched the inclusion criteria and were consecutively analysed (Tables 1 and 2). Nine of these are randomised controlled, 22 retrospective and 32 prospective studies. Comparing both groups, in 46 studies, conventional instruments were used, and in 27 studies, piezoelectric devices were used to perform the MSA (Tables 1 and 2).

**Table 1:** Overview on the event rate (with lower and upper limits, *z* value), weight and significance (*p* values) for conventional approach and random effect

Conventional	Event rate	Lower limit	Upper limit	*z* value	*p* value	Weight
Galindo-Moreno et al. 2007 [15]	0.005	0.000	0.076	− 3.726	0.000	0.445
Marchetti et al. 2007 [16]	0.010	0.001	0.143	− 3.218	0.001	0.443
Delibasi et al. 2013 [67]	0.043	0.006	0.252	− 3.023	0.003	0.753
van den Bergh et al. 1998 [5]	0.048	0.016	0.140	− 5.033	0.000	1.507
Krekmanov et al. 1995 [17]	0.071	0.030	0.160	− 5.527	0.000	1.870
Watzek et al. 1998 [18]	0.100	0.038	0.238	− 4.169	0.000	1.683
Zijderveld et al. 2008 [19]	0.102	0.059	0.171	− 7.153	0.000	2.394
Papa et al. 2005 [20]	0.105	0.054	0.197	− 5.726	0.000	2.162
Rickert et al. 2011 [73]	0.111	0.042	0.261	− 3.921	0.000	1.673
Rickert et al. 2013 [10]	0.111	0.042	0.261	− 3.921	0.000	1.673
Lindenmüller and Lambrecht 2006 [21]	0.112	0.063	0.191	− 6.462	0.000	2.343
Penarrocha-Diago et al. 2012 [22]	0.125	0.048	0.289	− 3.640	0.000	1.661
Kaptein et al. 1998 [23]	0.159	0.097	0.251	− 5.713	0.000	2.438
Cha et al. 2014 [24]	0.161	0.118	0.216	− 8.932	0.000	2.765
Tawil et al. 2001 [25]	0.167	0.071	0.343	− 3.285	0.001	1.791
Yilmaz et al. 2012 [26]	0.172	0.098	0.284	− 4.746	0.000	2.305
Cho et al. 2001 [27]	0.184	0.098	0.317	− 4.043	0.000	2.178
van den Bergh et al. 2000 [28]	0.200	0.093	0.379	− 3.037	0.002	1.894
Becker et al. 2008 [68]	0.204	0.154	0.265	− 7.779	0.000	2.791
Ewers et al. 2005 [29]	0.206	0.156	0.266	− 7.894	0.000	2.801
Aimetti et al. 2001 [30]	0.214	0.100	0.402	− 2.821	0.005	1.881
Hernández-Alfaro et al. 2008 [69]	0.219	0.184	0.259	− 11.435	0.000	2.934
Barone et al. 2008 [31]	0.231	0.076	0.522	− 1.829	0.067	1.346
Khoury et al. 1999 [3]	0.241	0.188	0.302	− 7.217	0.000	2.831
Barone et al. 2006 [32]	0.250	0.182	0.334	− 5.297	0.000	2.701
Raghoebar et al. 2001 [33]	0.258	0.200	0.327	− 6.230	0.000	2.805
Kim et al. 2011 [34]	0.259	0.129	0.453	− 2.391	0.017	1.948
Shlomi et al. 2004 [35]	0.274	0.184	0.387	− 3.714	0.000	2.533
Wannfors et al. 2000 [36]	0.275	0.159	0.432	− 2.738	0.006	2.228
Hallman et al. 2004 [37]	0.300	0.164	0.483	− 2.127	0.033	2.080
Bornstein et al. 2008 [38]	0.305	0.201	0.433	− 2.911	0.004	2.466
Ardekian et al. 2006 [39]	0.318	0.238	0.411	− 3.723	0.000	2.709
Kazancioglu et al. 2013 [40]	0.320	0.169	0.522	− 1.758	0.079	1.982
Raghoebar et al. 1999 [41]	0.321	0.249	0.403	− 4.129	0.000	2.775
Philippart et al. 2003 [42]	0.333	0.158	0.571	− 1.386	0.166	1.761
Scarano et al. 2015 [43]	0.333	0.131	0.624	− 1.132	0.258	1.455
Oh et al. 2011 [44]	0.343	0.276	0.416	− 4.085	0.000	2.830
Raghoebar et al. 1997 [45]	0.346	0.250	0.455	− 2.731	0.006	2.623
Jensen et al. 1994 [46]	0.352	0.274	0.438	− 3.307	0.001	2.764
Froum et al. 2013 [47]	0.375	0.240	0.532	− 1.564	0.118	2.320
Stricker et al. 2003 [48]	0.379	0.271	0.501	− 1.950	0.051	2.560
Levin et al. 2004 [49]	0.468	0.362	0.578	− 0.562	0.574	2.648
Schwartz-Arad et al. 2004 [4]	0.469	0.364	0.578	− 0.555	0.579	2.657
Papa et al. 2009 [50]	0.511	0.371	0.649	0.146	0.884	2.437
Kasabah et al. 2003 [6]	0.562	0.480	0.640	1.486	0.137	2.812
Krennmair et al. 2007 [51]	0.575	0.420	0.717	0.945	0.345	2.343
*Random*	*0.240*	*0.205*	*0.278*	*− 11.262*	*0.000*

**Table 2:** Overview on the event rate (with lower and upper limits, *z* value), weight and significance (*p* values) for piezosurgical approach and random effect

Piezoelectric	Event rate	Lower limit	Upper limit	*z* value	*p* value	Weight
Wallace et al. 2007 [52]	0.005	0.000	0.074	− 3.741	0.000	1.660
Sohn et al. 2010 [53]	0.008	0.001	0.054	− 4.817	0.000	2.712
Toscano et al. 2010 [54]	0.009	0.001	0.125	− 3.328	0.001	1.655
He et al. 2013 [55]	0.010	0.001	0.143	− 3.218	0.001	1.653
Stübinger et al. 2008 [56]	0.019	0.001	0.244	− 2.753	0.006	1.642
Kim et al. 2012 [57]	0.028	0.015	0.052	− 11.010	0.000	6.342
Blus et al. 2008 [58]	0.038	0.009	0.139	− 4.493	0.000	3.924
Scarano et al. 2015 [43]	0.038	0.002	0.403	− 2.232	0.026	1.616
Penarrocha-Diago et al. 2012 [22]	0.040	0.006	0.235	− 3.114	0.002	2.656
Delibasi et al. 2013 [67]	0.043	0.006	0.252	− 3.023	0.003	2.649
Moon et al. 1 (Moon et al. 2015) [74]	0.045	0.003	0.448	− 2.103	0.035	1.607
Moon et al. 2 (Moon et al. 2015) [70]	0.045	0.003	0.448	− 2.103	0.035	1.607
Vercellotti et al. 2001 [8]	0.048	0.007	0.271	− 2.924	0.003	2.642
Cortes et al. 2012 [59]	0.050	0.013	0.179	− 4.059	0.000	3.900
Stacchi et al. 2013 [60]	0.056	0.021	0.139	− 5.507	0.000	5.118
Stübinger et al. 2009 [61]	0.063	0.009	0.335	− 2.622	0.009	2.615
Gemiani et al. 2015 [76]	0.093	0.047	0.175	− 6.134	0.000	6.030
Felice et al. 2013 [71]	0.100	0.025	0.324	− 2.948	0.003	3.799
Rickert et al. 2011 [73]	0.111	0.042	0.261	− 3.921	0.000	5.018
Rickert et al. 2013 [10]	0.111	0.042	0.261	− 3.921	0.000	5.018
Sánchez-Recio et al. 2010 [62]	0.154	0.059	0.345	− 3.136	0.002	4.936
Sohn et al. 2011 [72]	0.164	0.091	0.279	− 4.711	0.000	6.189
Barone et al. 2013 [63]	0.167	0.055	0.409	− 2.545	0.011	4.406
Cassetta et al. 2012 [75]	0.175	0.086	0.324	− 3.726	0.000	5.745
Weitz et al. 2014 [64]	0.175	0.086	0.324	− 3.726	0.000	5.745
Barone et al. 2008 [31]	0.308	0.120	0.591	− 1.349	0.177	4.590
Corinaldesi et al. 2013 [65]	0.333	0.131	0.624	− 1.132	0.258	4.522
*Random*	*0.080*	*0.055*	*0.115*	*− 11.815*	*0.000*	

The forest plots generally show a higher perforation rate for conventional sinus lift when compared to piezosurgery (Tables 1 and 2, Figs. 2 and 3). It is obvious that studies with smaller sample size reveal higher 95% confidence intervals. The random effect for conventional sinus lift was 0.24 and for the piezo 0.08. This difference between piezo and conventional sinus lift was statistically highly significant with *p* < 0.001. (Figs. 2 and 3).

### Discussion

The current data show that there is a statistically significant less occurrence of perforation of the Schneiderian membrane when piezosurgery is used compared to conventional approach. The reason for this difference may be explained by the technical skills of piezoelectrical surgery. Piezoelectric devices are able to cut highly mineralized bone due to its surgical power which is three times higher than normal ultrasound and the variable modulations of the powerful piezoelectric handpiece with its functional frequency of 25 to29 kHz. Specifically designed osteotomy and osteoplasty inserts move with linear microvibrations (60 to 210 μm), which are ideal for the preservation of the Schneiderian membrane. Low frequency of ultrasonics and the sharp instruments cut mineralized tissue easier than soft tissue. Furthermore, it should be noted that near soft tissue, the cutting process is safer, while not using the intrinsic cutting and using a diamond-coated instrument [8].

This aspect may be especially crucial in MSA since the facial bone is mainly compact and the Schneiderian membrane rather thin and fragile. It could be shown that the mean Schneiderian membrane thickness is 1.13 mm [66]. Therefore, piezosurgery, with its gentle cutting process, is perfectly qualified for the maxillary sinus membrane elevation.

Though both techniques exist more than 20 years, only single studies could be found in which the incidence of membrane perforation was focused comparing both operative techniques. This was the rationale for our meta-analysis. Principally, there is a controversy in the literature concerning the use of piezosurgical devices for MSA. Torrella et al. showed a reduced risk for perforations of the sinus membrane while using ultrasound for lateral approach. They additionally mentioned the improved visibility and hygiene in the operating area and the controlled osseous incision [7]. Wallace et al. recorded a reduced membrane perforation rate, improved intraoperative visibility, reduced intraoperative bleeding and reduced surgical trauma. No perforation occurred during the antrostomy and the initial membrane elevation with piezoelectric inserts. However, using conventional hand instruments, seven membrane perforations occurred in the same study [52]. Stübinger et al. and Toscano et al. also reported on complications during the elevation with hand instruments, especially in delicate situations with underwood septa which have shown to be an additional risk for membrane perforation [54, 61]. In contrast to these studies, Barone et al. observed four membrane perforations in the group treated with piezosurgery and only three perforations in the group treated with conventional instruments. In this randomised controlled clinical trial comparing rotary instruments with a piezoelectric device during maxillary sinus floor elevation, no significant difference was observed between the two groups. The authors concluded that the major limitation of piezosurgery was the time factor. Cutting procedures were substantially longer compared with conventional osteotomy devices [31]. Rickert et al. assessed also in a randomised controlled trial the same issue. In their study, they found no differences in the occurrence of perforations of the sinus membrane during surgery between piezo and conventional approach. They concluded that piezosurgery showed no advantages over conventional rotating instruments. Furthermore, they mentioned that the result is strongly depending on the experience of the respective surgeon with one of the respective techniques [10]. Another randomised controlled trial of Scarano et al. found a statistically significant difference between the incidences of sinus membrane perforation in the two groups. Group 1 used a round oral surgery bur, and the elevation was completed with sinus lift instruments. Group 2 using an ultrasonic surgery created a lateral bony window with nasal suction technique and elevation by using standard sinus lift instruments. Group 1 presented four perforations of the membrane, and no perforation occurred in group 2 [43].

Atieh [11] found no significant difference in perforation risk. In these studies, occurred in the two groups of the RCTs are almost identical perforations. Maybe due to the fact that they included only one RS, while our study included 22, they see no deviation.

The review of Stacchi [12] also described a lower incidence of membrane perforation during piezosurgery (10.9%) than during conventional surgery (20.1%). These results are comparable with ours.

Geminiani [13] found a significantly lower incidence of membrane perforations by the meta-analysis of the retrospective studies. Such a difference was not detected during meta-analysis of the data collected from the randomised clinical trials. They describe that the differences are most likely due to the inherent limitations of retrospective studies that include biases in selection of control and exposure to risk variables. They say that RCTs should be considered the main, because these trials have the preferred design for assessing differences in the outcome of a systematic review.

While incorporating selected non-randomised and non-controlled prospective and retrospective studies, our current data show that there is less perforation of the Schneiderian membrane when using piezosurgery. This might be a weakness resulting from the inclusion of the non-controlled studies.

## Conclusions

The aim of the present study was to resume in a review the literature evaluating the incidence of sinus membrane perforation comparing conventional rotating instruments with piezoelectric devices. Since only scarce studies exist comparing both techniques directly, we decided to additionally include any study on MSA in which information on the applied technique, e.g. conventional or piezosurgery, was retrievable and additionally reporting on the incidence of membrane perforations. This procedure leads to heterogeneity of the collected data and accordingly included study designs. Obviously, a lot more studies with conventional approach than piezosurgery were included determining the incidence of membrane perforations. In order to reduce the resulting bias, forest plots were calculated and the weight was adjusted on the raw data, resulting in determination of the random effect. Another bias may affect the use of both conventional drilling for antrostomy and additionally piezosurgery for initial membrane elevation. The data were inconsistent in detailed information if combinations were used. Therefore, we decided to determine the piezosurgery group for both: single use for osteotomy and membrane elevation as well as for initial membrane elevation alone.

The present study may have a weakness, because membrane tear detection was not the primary focus and endpoint of many of our included studies. Many perforations might have been overlooked or not paid attention to this specific problem. The impact of different piezo tips on membrane perforation risk needs to be evaluated in further studies. With regard to our results, the present study showed that the weighted average incidence of perforation during MSA is 24% for rotating instruments and 8% for piezosurgery. These differences were statistically highly significant (*p* < 0.005). With regard to the presented results, piezosurgery can be recommended reducing the risk of membrane perforation during MSA. However, though this seems to be a reliable statement, it is not clear, whether combinations, e.g. osteotomy with rotating instruments and preparation of the membrane with piezosurgery, may be an alternative approach combining time efficiency with safety. The results suggest that piezosurgery was associated with a lower perforation rate. However, this statement is not reliable because of the inclusion of non-randomised and non-controlled studies as well as retrospective data. More RCTs focusing on membrane perforation are needed for a final conclusion.
